# Comparison of Current Techniques for Securing the Cystic Duct in Laparoscopic Cholecystectomy: A Systematic Review and Meta-Analysis

**DOI:** 10.7759/cureus.75787

**Published:** 2024-12-16

**Authors:** Lorenzo Verani, Yana Murateva, Neil Muscat, Sri Harsha Dintakurti, Shaneel Shah, Oddai Alkhazaaleh

**Affiliations:** 1 Surgery, Wrightington, Wigan and Leigh NHS Foundation Trust, Wigan, GBR; 2 Surgery, Imperial College Healthcare NHS Trust, Imperial College London, London, GBR; 3 Vascular Surgery, Manchester Royal Infirmary, Manchester University NHS Foundation Trust, Manchester, GBR; 4 Surgery, Liverpool University Hospital NHS Foundation Trust, Liverpool, GBR; 5 General Surgery, Manchester Royal Infirmary, Manchester University NHS Foundation Trust, Manchester, GBR; 6 General Surgery, Royal Bolton Hospital, Bolton NHS Foundation Trust, Bolton, GBR

**Keywords:** bile duct surgery, common bile duct, hem-o-lok, laparoscopic cholecystectomy complication, lap cholecystectomy

## Abstract

Laparoscopic cholecystectomy is a widely performed procedure, with securing the cystic duct being a critical step to prevent bile leakage. Traditional metal clips are commonly used, but alternative methods, such as non-absorbable polymer clips, absorbable clips, sutures, and ultrasonic shears, are also utilized. This systematic review and meta-analysis evaluates the safety and efficacy of various cystic duct securing techniques. Following PRISMA guidelines, a comprehensive search was conducted across the EMBASE, MEDLINE, and Cochrane databases without date restrictions. Eligible studies compared different techniques for cystic duct closure in laparoscopic cholecystectomy, focusing on bile leakage as a primary outcome. Data extraction and synthesis were performed using a binary random-effects model. Meta-analyses were conducted for absorbable clips, sutures, hem-o-lok clips, and ultrasonic devices compared to standard metal clips. A total of 26 studies met the inclusion criteria. Absorbable polymer clips demonstrated a statistically significant reduction in postoperative bile leakage compared to metal clips (OR 0.159; 95% CI 0.031-0.818; P=0.028). In contrast, no significant differences were observed when comparing metal clips with suture ties (OR 0.459, 95% CI 0.139-1.522, *P*=0.203), non-absorbable clips (OR 0.166, 95% CI 0.025-1.109, *P*=0.064), or the clipless technique with a harmonic device (OR 1.332, 95% CI 0.425-4.169, *P*=0.623).

## Introduction and background

Laparoscopic cholecystectomy (LC) is the minimally invasive surgical removal of the gallbladder. It is the gold-standard treatment for gallstone disease, a common condition that affects around 10-15% of adults in Western countries [1]. Worldwide, it is estimated that millions of these procedures are performed annually, and in the UK alone, about 70,000 laparoscopic cholecystectomies are performed each year [2]. This high frequency is due to gallstones’ prevalence, often requiring surgical intervention when symptomatic, or when complications like cholecystitis, biliary colic, or pancreatitis arise.

Complications of laparoscopic cholecystectomy

While LC is generally considered safe, it is associated with several complications. The most significant are bile leakage and bile duct injury (BDI), which can have severe consequences, such as infection, and, in rare cases, mortality. The incidence of BDI is estimated at 0.3-0.6% of cases, a higher rate than in traditional open cholecystectomy. Other complications include bleeding, surgical site infections, and retained stones in the bile duct [3]. While most patients recover uneventfully, around 1-2% experience significant postoperative complications, some of which require additional surgeries or hospitalizations.

Morbidity and NHS costs related to complications

Complications from LC pose substantial morbidity for patients and financial burdens for healthcare systems like the NHS. For example, a BDI can necessitate additional interventions, such as endoscopic stenting, percutaneous drainage, or even liver transplantation in severe cases [4]. Similarly, a post-operative bile leak due to failure to secure the common bile duct might warrant additional intervention. The costs associated with these treatments can be substantial, with estimates suggesting that treating a bile duct injury can cost the NHS between £5,000 and £100,000 per patient [5]. The long-term management of complications also affects patients' quality of life, with some experiencing chronic pain, infections, or bile duct stricture, which may require ongoing care. This increase in morbidity translates to increased healthcare costs and a prolonged recovery period, often resulting in lost productivity and extended patient suffering.

Methods of securing the cystic duct

The cystic duct must be securely closed during LC to prevent bile leakage, which is a primary cause of postoperative complications. Several methods are used to secure the cystic duct, including clips (usually titanium or absorbable polymer), sutures, staplers, and energy-based sealing devices [6]. Titanium clips, which are easy to apply and generally secure, are the most commonly used method. Absorbable clips have also gained popularity for their reliability and reduced long-term foreign body presence. In cases of complex anatomy or a wide cystic duct, surgeons may opt for endoloops or suturing. However, these methods can be more technically challenging and time-consuming [7].

Evidence on the best cystic duct-securing method

Research comparing methods of securing the cystic duct is ongoing. However, titanium clips remain the most widely used and studied option due to their simplicity and efficacy in most cases [6,8]. Absorbable clips are shown to be similarly effective with the added benefit of not leaving permanent foreign material [9]. Studies have demonstrated that both titanium and absorbable clips have similar rates of complication, including bile leakage and clip slippage. Energy-based devices, while useful in some settings, are generally less preferred due to the increased risk of thermal injury to adjacent structures [10]. In complex cases with large or friable cystic ducts, endoloops and sutures have been shown to be effective and may reduce bile leakage risk. However, they require more surgical expertise.

In summary, laparoscopic cholecystectomy is a common and generally safe procedure for gallstone disease, with bile duct injury being the most significant complication. While titanium clips remain the standard for securing the cystic duct, absorbable clips and, in some cases, suturing, are viable alternatives. Ongoing research continues to refine the best practices for minimizing complications and optimizing outcomes for patients undergoing this essential surgery.

The aim of this up-to-date systematic review and meta-analysis was to compare the incidence of postoperative bile leakage in laparoscopic cholecystectomies across different methods of cystic duct closure, specifically comparing traditional metal clips to absorbable clips, suture materials, non-absorbable polymer clips, and ultrasonic shears.

## Review

Methods

Literature Search Strategy

A comprehensive online search of the EMBASE, MEDLINE and Cochrane databases was conducted. The following search was performed - ​​Laparoscopic cholecystectomy AND (clip OR lock OR hem-o-lok OR tie OR knot OR suture tie OR loop). There were no date criteria for exclusion.

The review of the studies for the systematic review was conducted following the PRISMA (Preferred Reporting Items for Systematic Reviews and Meta-Analyses) guidelines. The review protocol was registered with the International Prospective Register of Systematic Reviews, PROSPERO, on 30 October 2024 (CRD42024603983). 

Inclusion and Exclusion Criteria

All studies were imported into Covidence for a preliminary abstract review and identification of any duplicates. Studies were considered eligible for inclusion if the patients underwent laparoscopic cholecystectomy as the intervention, the studies compared at least two different methods for cystic duct closure, and one of the primary outcomes the study examined was the rate of bile leakage as a post-operative complication (bile leaks from the accessory duct of Luschka were disregarded). The review included devices such as hem-o-lok, metal clips, absorbable clips, locking polymer, bipolar, monopolar, and harmonic scalpel, as well as simple ligation of the cystic duct with sutures. Our exclusion criteria were animal studies, ex-vivo studies, case reports, case series, and those articles which were not available in English or for which official English translations were not available. Efforts were made to retrieve all relevant studies identified in the search. In cases where full-text articles could not be obtained, requests were made to two different institutional libraries. If both libraries were unable to locate the full text, the study was excluded due to unavailability. These exclusions are documented in the PRISMA flowchart.

Study Selection and Data Extraction

All of the studies were reviewed independently by two authors (LV and YM) with any disputes having been resolved by a third author. Following the abstract selection, a full-text review was conducted and the rates of bile leak were extracted from all of the eligible papers for meta-analysis. This was done using the Covidence systematic review software (Veritas Health Innovation, Melbourne, Australia) [11]. 

Data Synthesis

Data were synthesized using the OpenMeta Analyst (Brown University School of Public Health, Providence, RI) software [12]. The analysis involved utilizing a binary fixed-effects model with a generic inverse variance function applied to account for cases of moderate to high heterogeneity and utilizing an odds ratio (OR) on the dichotomous data. The primary outcomes were reported on a forest plot with 95% confidence intervals (CIs).

Results

**Figure 1:**
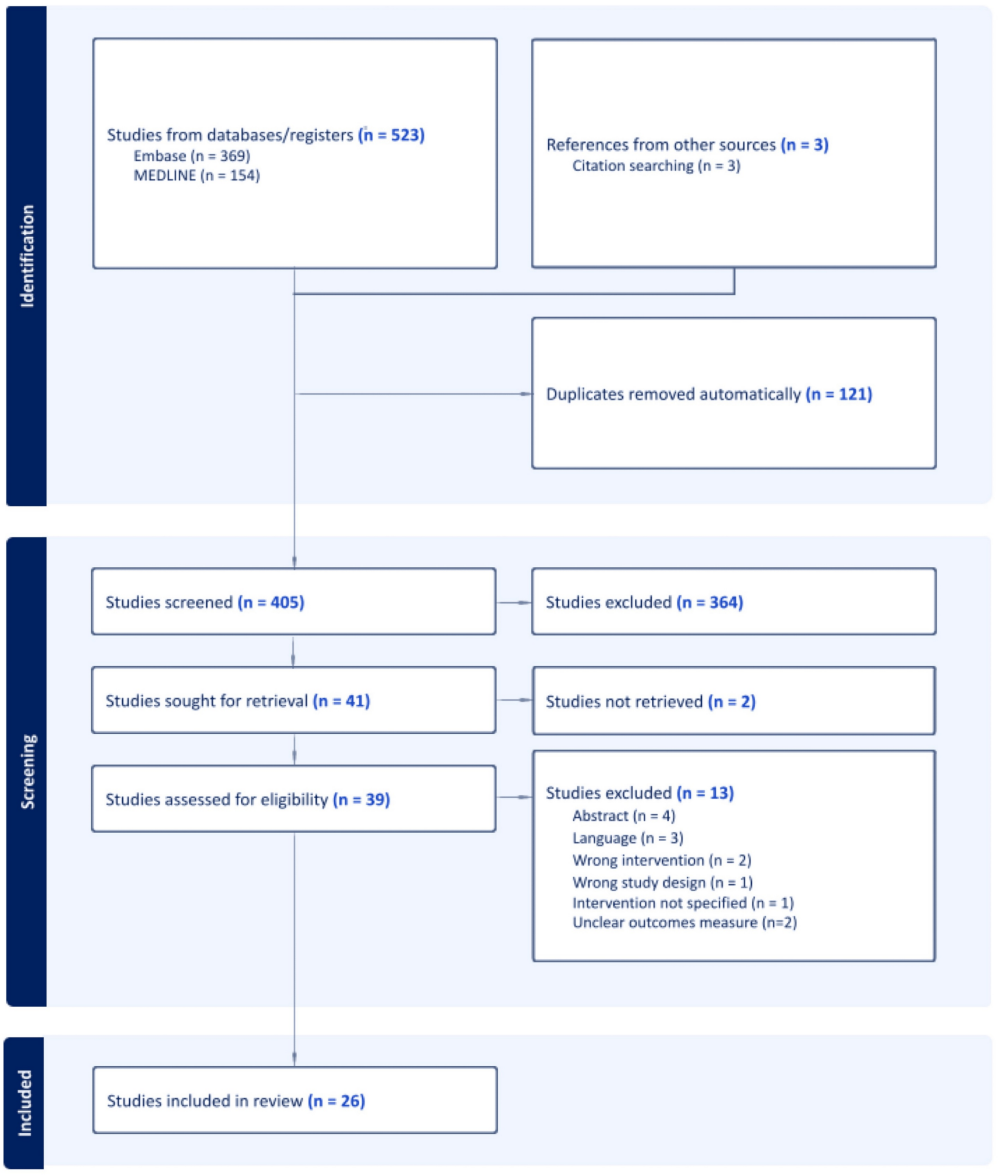
PRISMA flowchart PRISMA (Preferred reporting items for systematic reviews and meta-analysis) flow diagram detailing the search and selection processes applied during the literature review. Twenty-six studies were included in the final analysis [8,13-37]. Two studies were excluded as the full-text versions were unavailable [38,39].

After adhering to PRISMA recommendations, 26 papers were identified as eligible for inclusion in our systematic review (Figure 1).

Studies Selected for the Final Analysis

A total of twenty-six studies were deemed eligible for inclusion in the qualitative analysis following full-text review. Characteristics of the studies are included in Table 1. Of these studies, 21 reported comparisons with metal clips and thus were included in the meta-analysis [8,13-37].

**Table 1:** Included studies characteristics Table summarising studies included in systematic review [8,13-37]. PDS=Polydioxanone Suture

First author, year of publication	Country	Study type (population)	Compared closure methods
Hawasli, 1994 [13]	USA	Randomised controlled trial (n=50)	Metal clips vs absorbable clips
Bencini et al., 2003 [14]	Italy	Retrospective cohort (n=690)	Metal clips vs absorbable clips
Yano et al., 2003 [15]	Japan	Retrospective cohort (n=772)	Metal clips vs absorbable clips
Hüscher et al., 2003 [16]	Italy	Prospective non-randomised clinical trial (n=461)	Harmonic shears vs harmonic scissors + endo-loop
Rohatgi et al., 2006 [17]	UK	Retrospective cohort (n=490)	Metal clips vs absorbable clips
Bessa et al., 2007 [18]	Egypt	Randomised controlled trial (n=120)	Metal clips vs harmonic scalpel
Kandil et al., 2009 [19]	Egypt	Randomised controlled trial (n=140)	Metal clips vs harmonic scalpel
Kavlakoglu et al., 2010 [20]	Turkey	Prospective case control (n=60)	Metal clips vs harmonic scalpel
Redwan, 2010 [21]	Egypt	Randomised controlled trial (n=160)	Metal clips vs harmonic scalpel
Matsui et al., 2012 [22]	Japan	Retrospective cohort (n=1101)	Absorbable clips vs Hem-o-lok clips vs metal clips vs endo-loop vs suture vs endolinear cutter
Mirani et al., 2012 [23]	Pakistan	Retrospective cohort (n=183)	Harmonic scalpel vs monopolar diathermy
Wills et al., 2013 [24]	USA	Retrospective cohort (n=208)	Metal clips vs harmonic scalpel
Yang et al., 2014 [25]	China	Retrospective cohort (n=1363)	Metal clips vs absorbable clips
Baloch et al., 2015 [26]	Pakistan	Randomised controlled trial (n=86)	Metal clips vs harmonic scalpel
Leo et al., 2016 [27]	India	Randomised controlled trial (n=293)	Metal clips vs sutures
Abdelhady et al., 2017 [28]	Kuwait	Randomised controlled trial (n=60)	Metal clips vs harmonic scalpel
Bali et al., 2018 [29]	India	Randomised controlled trial (n=120)	Metal clips vs sutures (silk)
Rajnish et al., 2018 [30]	India	Prospective, parallel arm, non-randomised controlled trial (n=40)	Metal clips vs harmonic scalpel
Singal et al., 2018 [31]	India	Randomised controlled trial (n=140)	Metal clips vs sutures (silk)
Donkervoort et al., 2020 [32]	Netherlands	Retrospective cohort (n=4359)	Metal clips vs PDS loops
Madhavan et al., 2021 [8]	India	Retrospective cohort (n=1496)	Metal clips vs Hem-o-lok clips
Poillucci et al., 2021 [33]	Italy	Retrospective cohort (n=154)	Metal clips vs Hem-o-lok clips
Soomro et al., 2022 [34]	The Kingdom of Saudi Arabia	Randomised controlled trial (n=100)	Metal clips vs endo-loop
Yeni et al., 2022 [35]	Turkey	Prospective observational (n=114)	Silk sutures vs Hem-o-lok clips
Chidambaram et al., 2023 [36]	India	Cross-sectional (n=60)	Metal clips vs intracorporeal ligation
Jayalal et al., 2023 [37]	India	Randomised controlled trial (n=50)	Metal clips vs bipolar diathermy

Bile Leak With Absorbable Polymer Clips Compared to Metal Clips

**Figure 2:**
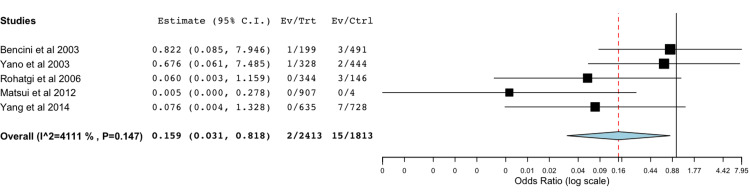
Bile leak with absorbable polymer clips compared to metal clips A forest plot depicting rates of bile leak which were found to be significantly higher with the metal clip group compared to the absorbable polymer clip group [14,15,17,22,25]. Odds ratio 0.159 (0.031, 0.818),  *P*-value 0.028.

A total of five studies were included in the meta-analysis of data comparing rates of postoperative bile leak with absorbable polymer clips compared to standard metal clips (Figure 2) [14,15,17,22,25]. The statistical analysis utilizing a binary random-effects model was of low heterogeneity and found a significant difference between the two groups with a *P*-value of 0.028. 

Bile Leak With Suture Material Compared to Metal Clips 

**Figure 3:**
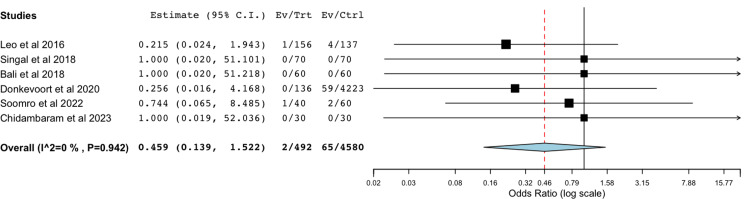
Comparison of bile leak rates between suture group and metal clip group Forest plot comparing the rates of bile leak between the suture group and the metal clip group [27,29,31,32,34,36]. Overall, no statistically significant difference was found, with an odds ratio of 0.459 (95% CI: 0.139–1.522, *P*=0.203).

A total of six studies were included in the meta-analysis of data comparing rates of postoperative bile leak with suture material compared to standard metal clips (Figure 3) [27,29,31,32,34,36]. The statistical analysis utilizing a binary random-effects model was of low heterogeneity and found no significant difference between the two groups with a *P*-value of 0.203.

Bile Leak With Non-absorbable Polymer Clips Compared to Metal Clips

**Figure 4:**
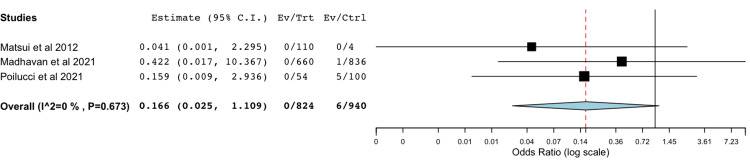
Comparison of bile leak rates between non-absorbable polymer clip and metal clip Forest plot comparing the rates of bile leak between the non-absorbable polymer clip group and the metal clip group [8,22,33]. No statistically significant difference was found, with an odds ratio of 0.166 (95% CI: 0.025–1.109, *P*=0.064).

A total of three studies were included in the meta-analysis of data comparing rates of postoperative bile leak with non-absorbable polymer clips compared to standard metal clips (Figure 4) [8,22,33]. The statistical analysis utilizing a binary random-effects model was of low heterogeneity and did not find a significant difference between the two groups with a *P*-value of 0.64. 

Bile Leak With Harmonic Scalpel Compared to Metal Clips

**Figure 5:**
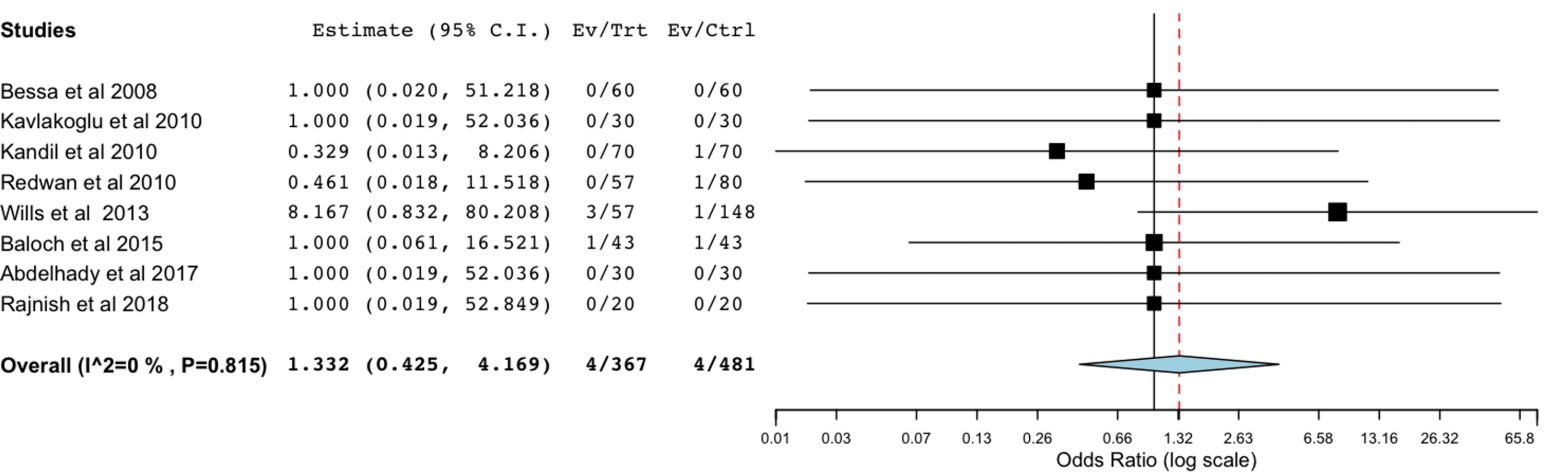
Comparison of bile leak rates between clipless tecnique and metal clip Forest plot comparing the rates of bile leak between the harmonic scalpel group and the metal clip group [18-21,24,26,28,30]. Overall no statistical difference was found, odds ratio of 1.332 (95% CI: 0.425–4.169, *P*=0.623).

A total of eight studies were included in the meta-analysis of data comparing rates of postoperative bile leak with a harmonic scalpel compared to standard metal clips (Figure 5) [18-21,24,26,28,30]. The statistical analysis utilizing a binary random-effects model was of low heterogeneity and did not find a significant difference between the two groups with a *P*-value of 0.623. 

Discussion

The main finding of the systematic review and meta-analysis is that absorbable clips are associated with a lower incidence of postoperative bile leakage compared to standard practice metal clips with a statistically significant reduction of 84% (OR 0.159 (0.031, 0.818), *P*-value 0.028). The other securing methodologies - sutures, hem-o-lok, and harmonic scalpel - showed no statistically significant difference in the rates of bile leakage compared to metal clips.

Absorbable Clips

The observed reduction in bile leaks with absorbable clips could be attributed to several factors. While metal clips have traditionally been the mainstay in laparoscopic cholecystectomy, they carry certain risks, particularly related to clip slippage or ductal necrosis caused by thermal injury. An ex-vivo study previously suggested that metal clips have a lower dislocation force compared to absorbable clips [40]. Additionally, case reports document complications such as clip migration, leading to conditions like common duct stone formation or abdominal pain [41,42]. In our analysis, the lower incidence of bile leaks with absorbable clips may stem from advantages such as improved tactile feedback during application and a more secure locking mechanism.

Clipless Cholecystectomy

To the best of our knowledge, this is the first meta-analysis investigating the outcomes of clipless cholecystectomy with ultrasonic shears. This technique has been increasingly studied in India, Pakistan, and Egypt, and multiple high-quality prospective studies have been published [21,26,37].

The use of a single instrument throughout the procedure is associated with shorter operative times and potential cost savings. However, the safety profile remains under debate. Ex-vivo studies have yielded mixed results when comparing the cystic duct and gallbladder bursting pressures between harmonic shears, electrocautery, and metal clips [43,44]. In our meta-analysis, we found no statistically significant difference in bile leaks (OR 1.33 (0.425,4.169, p=0.623)) which may be due to the low incidence of this complication and the limited statistical power in our analysis. Further large-scale studies are needed to establish the safety of this approach before its widespread adoption. Notably, one study converted three patients from ultrasonic shears to metal clips due to insufficient ex-vivo burst pressure, possibly underestimating bile leakage rates in the former group [24].

Surgical Ties

We also present the most comprehensive review to date comparing metal clips and surgical ties for cystic duct closure, incorporating six new prospective studies. Collectively, these studies suggest no significant difference in postoperative bile leak rates between these two techniques [27,29,31,32,34,36].

Non-absorbable Polymer Clips

Currently, definitive conclusions regarding hem-o-lok vs metal clip could not be drawn due to the paucity of published data on the issue. However, our results are promising and point towards a better safety profile than metal clips. Further studies including metal clips could also allow for an indirect comparison, evaluating the safety of hem-o-lok compared to absorbable clips.

## Conclusions

Absorbable clips show a significant advantage over metal clips in reducing postoperative bile leakage, making them a valuable alternative for cystic duct closure. Similarly, non-absorbable polymer clips, such as hem-o-lok clips, appear to be promising. However, additional studies are needed to achieve statistical significance. The use of ultrasonic clipless techniques also warrants further rigorous investigation to establish their safety and efficacy before they can be adopted into routine clinical practice.
